# Individualized Prediction of Recurrence Following Uterine-Preserving Pelvic Organ Prolapse Repair Using Internally Validated Machine Learning Models

**DOI:** 10.3390/jcm15166366

**Published:** 2026-08-18

**Authors:** Shenhav Malul, Henry H. Chill, Rami Yosef, Talya Miron-Shatz, David Shveiky

**Affiliations:** 1 Department of Business Administration, Guilford Glazer Faculty of Business and Management, Ben-Gurion University of the Negev, Beer Sheba 8410501, Israel; shenhavo@gmail.com (S.M.);; 2 Division of Urogynecology, Department of Obstetrics and Gynecology, Hadassah Medical Organization and Faculty of Medicine, Hebrew University of Jerusalem, Jerusalem 9112001, Israel; 3 Faculty of Business Administration, Ono Academic College, Kiryat Ono 5545001, Israel

**Keywords:** pelvic organ prolapse, uterine preservation, apical prolapse, pelvic organ prolapse repair

## Abstract

**Background/Objectives**: Approximately 11–20% of women affected by pelvic organ prolapse (POP) will undergo surgical intervention during their lifetime and nearly one-third of these women will require an additional procedure due to recurrence or failure of the initial repair. This study aimed to identify risk factors for failure and to develop models to predict subjective, anatomical, and composite failure after primary uterine-preserving POP surgery. **Methods**: We performed a retrospective cohort study of women undergoing primary uterine-preserving POP repair at a tertiary academic medical center between 2010 and 2024. Failure outcomes were defined as: subjective failure (patient-reported prolapse symptoms), anatomical failure (prolapse beyond the hymen during exam), and composite failure (subjective and/or anatomical failure and/or reoperation for prolapse). Risk factors were evaluated using univariable and multivariable logistic regression with odds ratios and 95% confidence intervals. Prediction models were trained in Python and internally validated using bootstrap optimism correction (500 resamples). Discrimination, overall accuracy, and calibration were evaluated. **Results**: Among 277 women, subjective failure occurred in 36 (13.0%), anatomical failure in 24 (8.7%), and composite failure in 46 (16.6%). In multivariable risk-factor models, larger genital hiatus was associated with higher odds of failure across endpoints and posterior compartment descent was independently associated with anatomical and composite failure. After bootstrap optimism correction, discrimination remained high for the best-performing models. **Conclusions**: In this single-center cohort, independent risk factors for failure after primary prolapse surgery were identified and prediction models showed strong internal discrimination. However, calibration limitations necessitate recalibration and external validation before clinical implementation.

## 1. Introduction

Pelvic floor dysfunction (PFD) encompasses a spectrum of debilitating conditions affecting women, including pelvic organ prolapse (POP), urinary incontinence, voiding dysfunction, and defecatory disorders [1]. These conditions impose a substantial burden on affected individuals and healthcare systems, with US prevalence estimates indicating that over one-third of parous women experience at least one PFD symptom by midlife [2].

Initial management emphasizes conservative approaches such as pelvic floor muscle training and pessary use; however, approximately 200,000 US women undergo POP surgery annually, with native-tissue repairs favored for their durability in select cases despite elevated recurrence risks [3].

Recurrence rates following primary surgery range from 13 to 30% at 1–2 years, varying by definition (subjective symptoms, anatomical stage ≥ 2, or reoperation) and are influenced by patient factors (age, parity, obesity), anatomical features (prolapse stage, levator integrity), and procedural elements (compartment addressed, concomitant repairs) [4,5]. Key risk factors include advanced preoperative prolapse, levator ani avulsion, enlarged genital hiatus, and posterior/apical defects, yet these predictors account for limited prognostic variance in isolation [6,7,8].

Despite recognition of these risks, validated tools for individualized recurrence prediction remain scarce, limiting preoperative counseling, surgical planning, and tailored surveillance [9]. Existing nomograms demonstrate moderate discrimination (c-statistic 0.70–0.85) but suffer from overfitting, poor calibration in external cohorts, and reliance on imaging or specialized metrics unavailable in routine practice [10,11]. Machine learning approaches, leveraging routinely collected POP-Q data, hold potential to enhance accuracy but require rigorous internal validation to mitigate optimism bias prior to clinical deployment [12].

Uterine-preserving procedures during POP repair are a viable surgical option promoted in recent years by both caregivers and patients. Advantages include shorter operative time, reduced intraoperative blood loss, and shorter hospital stay compared to apical prolapse repair involving hysterectomy [13,14]. However, data regarding risk factors for recurrence within this specific group is sparse.

The aim of the current study was to (1) delineate independent risk factors for subjective, anatomical, and composite POP recurrence following primary uterine-preserving apical prolapse repair and (2) derive a bootstrap-validated machine learning prediction model using preoperative/perioperative variables from a real-world cohort. These models aim to establish a foundation for individualized risk stratification; prospective external validation and recalibration are required before clinical deployment.

## 2. Materials and Methods

### 2.1. Study Design and Setting

We performed a retrospective cohort study at a tertiary university-affiliated teaching hospital. The study included women who underwent primary uterine-preserving apical prolapse repair between 2010 and 2024.

### 2.2. Participants

Eligible patients were women diagnosed with POP, including apical prolapse, who underwent primary uterine-preserving surgical repair. Patients with prior pelvic surgery for prolapse/incontinence were excluded. Procedures were performed as part of routine clinical care and included laparoscopic uterosacral ligament suspension, sacrospinous hysteropexy, vaginal colposuspension utilizing the Uphold Lite Mesh System (Boston Scientific, Springfield, MA, USA) and laparoscopic sacrohysteropexy. Concomitant procedures were performed as indicated and included anterior and posterior colporrhaphy, and mid-urethral sling procedures for stress incontinence.

### 2.3. Data Sources and Variables

Data were obtained from electronic medical records and operative reports. Preoperative variables included demographics, past medical, surgical and obstetric history (e.g., parity and delivery characteristics), lifestyle factors (smoking, sexual activity), menopausal status, and POP-Q measurements (Aa, Ba, Ap, Bp, C, D, genital hiatus [GH], perineal body [PB], and total vaginal length [TVL]) recorded using standardized POP-Q terminology. Operative variables included apical procedure type and the performance of concomitant procedures (e.g., anterior cystocele repair when applicable).

### 2.4. Outcomes

Three binary outcomes were defined: (1) Subjective failure: presence of patient-reported prolapse symptoms at follow-up. (2) Anatomical failure: evidence of prolapse beyond the hymen on physical examination. (3) Composite failure: subjective and/or anatomical failure and/or documented reoperation for prolapse. Follow-up routine care included visits at approximately 6 and 12 months postoperatively and annually thereafter when available. Given the variable follow-up duration across the 2010–2024 study period, recurrence was analyzed as a binary outcome rather than using time-to-event methods; this approach does not account for differences in observation time between patients and should be considered a limitation when interpreting absolute failure rates.

### 2.5. Statistical Analysis

Baseline characteristics were compared between outcome groups for each endpoint. Categorical variables were assessed using the chi-square test (or Fisher’s exact test when expected cell counts were small), and continuous variables were evaluated using the independent-samples *t*-test. Corresponding *p*-values are reported for descriptive comparisons.

### 2.6. Risk-Factor Models

Risk factors for each endpoint were evaluated using logistic regression. We estimated univariable models for each candidate predictor and a multivariable model incorporating the selected covariates. Effects are reported as odds ratios (ORs) with 95% confidence intervals (CIs).

### 2.7. Prediction Models

**Figure A1:**
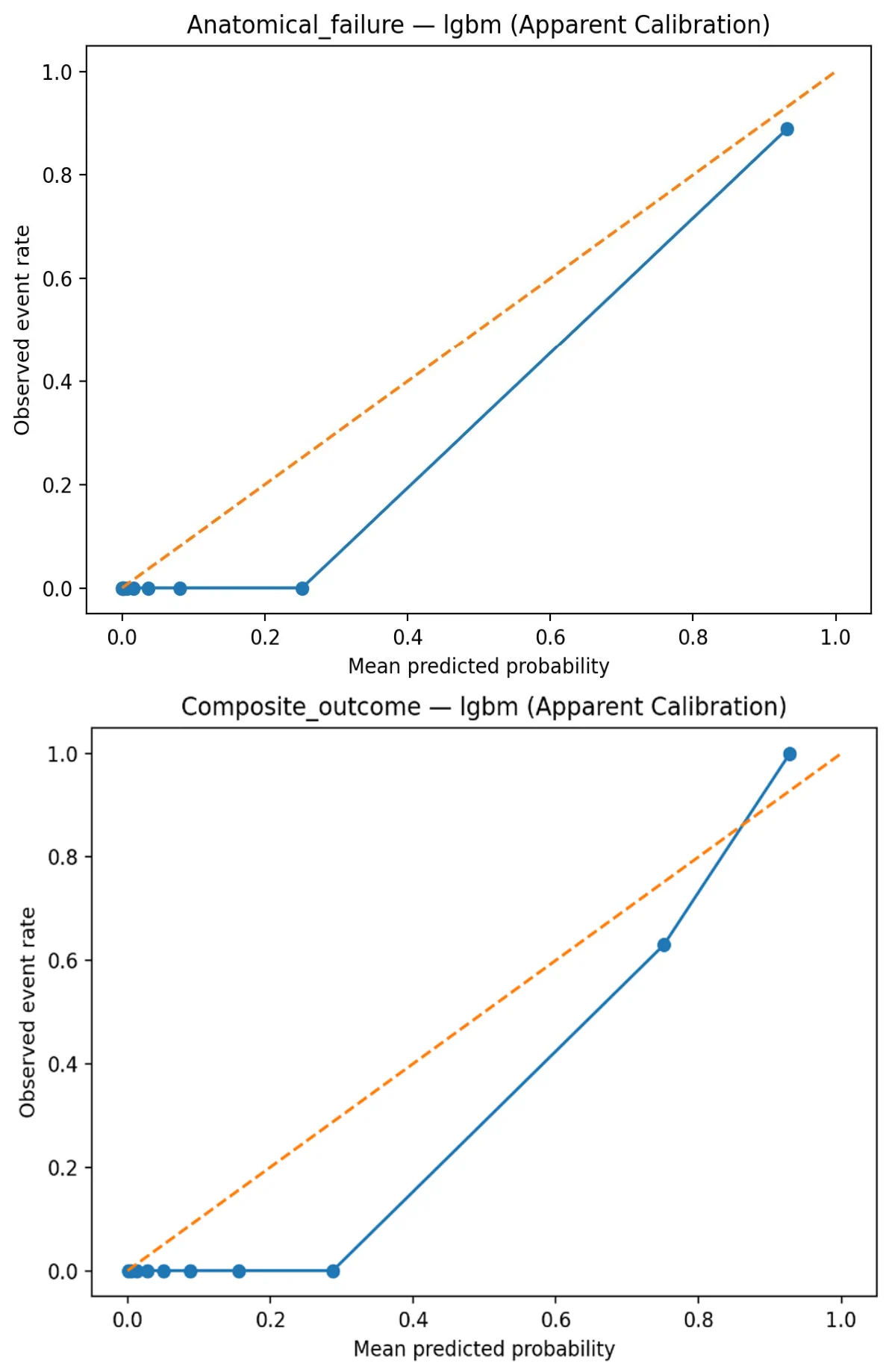

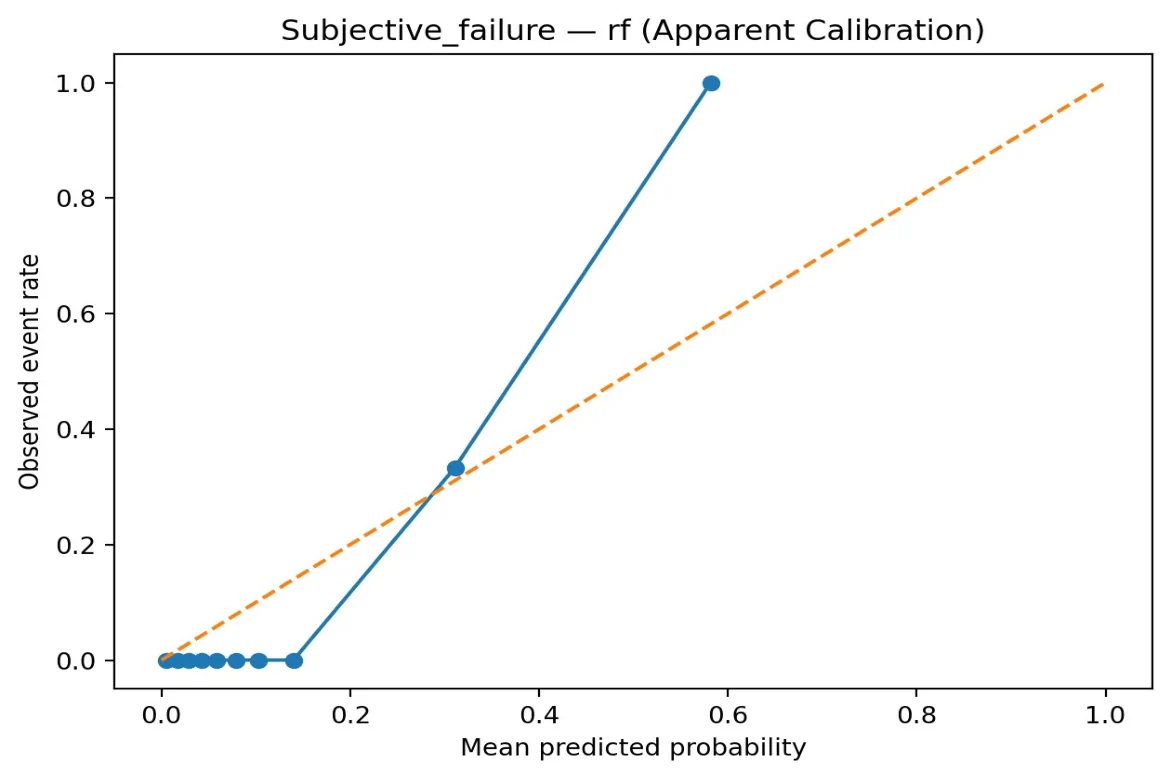
Apparent (in-sample) calibration plots for the three selected models: anatomical failure (LightGBM), composite outcome (LightGBM), and subjective failure (random forest). In each panel, the solid blue line with filled circles shows the observed event rate within bins of predicted risk plotted against the mean predicted probability of each bin, and the orange dashed diagonal line represents perfect calibration (predicted probability equal to observed event rate). Deviations of the blue curve from the diagonal indicate miscalibration: segments below the diagonal reflect overestimation of risk, whereas segments above it reflect underestimation.

**Figure A2:**
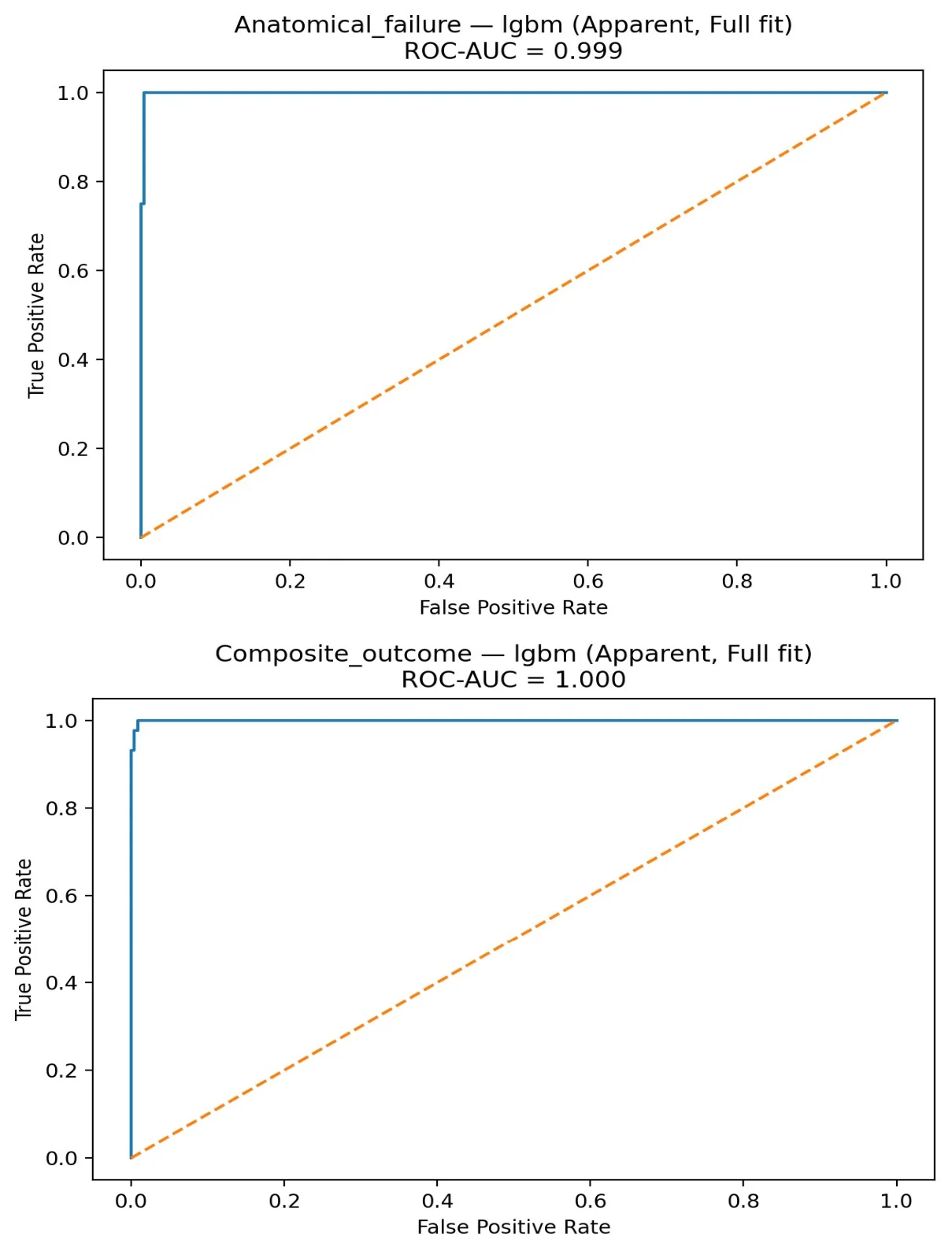

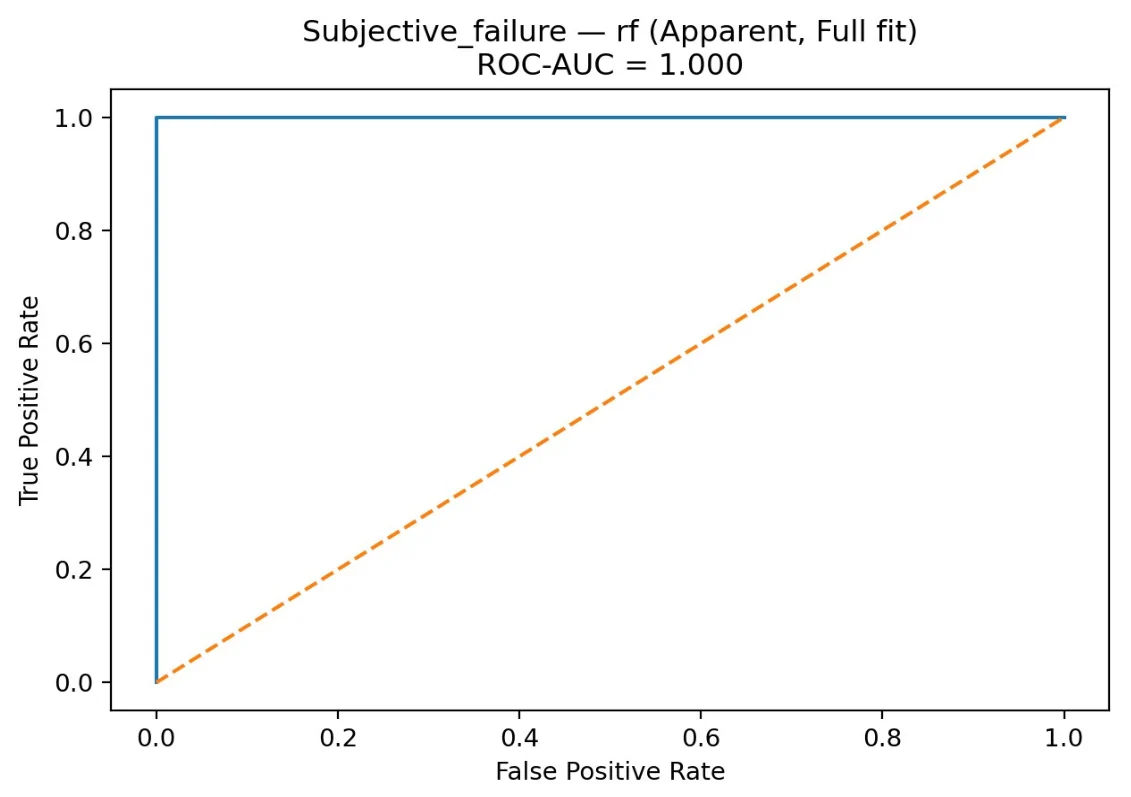
Apparent (full-fit) receiver operating characteristic (ROC) curves for the three selected models: anatomical failure (LightGBM; ROC-AUC = 0.999), composite outcome (LightGBM; ROC-AUC = 1.000), and subjective failure (random forest; ROC-AUC = 1.000). In each panel, the solid blue line is the ROC curve, plotting the true positive rate against the false positive rate across all classification thresholds, and the orange dashed diagonal line represents a random classifier with no discriminative ability (AUC = 0.5). Because these curves were derived from models fitted and evaluated on the full dataset without optimism correction, they overestimate discrimination relative to the internally validated performance reported in Table 7.

For each endpoint, prediction models were trained in Python 3.10 using penalized logistic regression, random forest, and gradient boosting (LightGBM). Internal validation was performed using bootstrap resampling (500 iterations with replacement) to estimate optimism-corrected performance. In each bootstrap sample, the full modeling pipeline—including feature preprocessing and, where applicable, hyperparameter selection—was refitted and evaluated both within the resampled dataset and in the original cohort to quantify overfitting. Missing covariate values were handled using median imputation for continuous variables and mode imputation for categorical variables; imputation parameters were estimated within each bootstrap resample to prevent information leakage. The optimism estimate, defined as the mean difference between bootstrap and original sample performance, was subtracted from the apparent performance to obtain corrected estimates. Model performance was summarized using AUC and PR-AUC for discrimination, Brier score for overall accuracy, and calibration intercept/slope and calibration curves. Calibration figures and model performance summaries generated in Python were incorporated into the manuscript from the prespecified project output directory (Figure A1 and Figure A2).

## 3. Results

Between 2010 and 2024, a total of 277 women met the inclusion criteria and underwent primary uterine-preserving POP repair. The mean age at surgery was 60.4 years (*SD* 10.0), and the mean BMI was 26.0 kg/m^2^ (*SD* 4.1). Surgical procedures comprised laparoscopic uterosacral ligament suspension (LUSLS), sacrohysteropexy, sacrospinous hysteropexy (SSL), and vaginal mesh repair using the Uphold system, with LUSLS representing the most common approach. Concomitant procedures were frequently performed, including anterior colporrhaphy, posterior repair, and mid-urethral sling placement. Postoperative follow-up was conducted at 6 and 12 months and annually thereafter as part of routine clinical care.

Subjective failure was assessed in 269 patients. As shown in Table 1, 36 patients (13.4%) experienced subjective failure, compared with 233 patients (86.6%) who did not. With respect to demographic characteristics, the mean BMI differed significantly between patients with and without subjective failure (*p* = 0.029). No medical comorbidities or prior delivery characteristics differed significantly between groups. For pelvic measurements, no preoperative or intraoperative attributes differed significantly between groups.

Anatomical failure was assessed in 268 patients. As shown in Table 2, 24 patients (9%) experienced anatomical failure, compared with 244 patients (91%) who did not. With respect to demographic characteristics, no attributes differed significantly between patients with and without anatomical failure. Regarding medical comorbidities, hyperthyroidism differed significantly by anatomical outcome (*p* = 0.012). For pelvic measurements, patients with anatomical failure had larger preoperative values for points C (*p* = 0.003), GH (*p* < 0.001), TVL (*p* < 0.001), Ap (*p* = 0.01), and Bp (*p* < 0.001) compared with patients without anatomical failure. No other attributes differed significantly between groups.

Composite failure was assessed in 270 patients. As shown in Table 3, 46 patients (17%) experienced composite failure, compared with 224 patients (83%) who did not. No demographic characteristics, medical comorbidities, or delivery characteristics differed significantly between groups. For pelvic measurements, patients with composite failure had larger preoperative values for points C (*p* = 0.05), GH (*p* = 0.005), TVL (*p* = 0.007), and Bp (*p* = 0.002) compared with patients without composite failure. No other attributes differed significantly between groups.

### 3.1. Risk Factors for Recurrence Following Primary Surgical Correction of Pelvic Organ Prolapse

We evaluated risk factors for distinct failure endpoints: subjective, anatomical, and composite, following primary POP surgery using logistic regression models and report effects as odds ratios (ORs). We estimated univariable models for each candidate predictor and a multivariable model incorporating the selected covariates. Candidate predictors were drawn from three prespecified domains: (1) demographic and anthropometric characteristics (e.g., age and BMI); (2) factors previously identified in the literature as significant predictors of failure after primary POP surgery; and (3) variables demonstrating statistically significant differences between patients who experience failure and those who do not. We acknowledge that the inclusion of criterion (3) introduces outcome-driven variable selection, which may constitute data leakage and inflate apparent model performance; this should be considered a limitation when interpreting results. Predictors with empty cells (zero observations in at least one outcome group) were excluded from regression analyses to ensure estimability and stable inference. Because no failures occurred among smokers, smoking was excluded due to complete separation (zero events), which precluded reliable model estimation.

Given the modest event counts (anatomical failure: 24 events; subjective failure: 36 events; composite failure: 46 events), the events-per-variable (EPV) ratio in the full multivariable models was limited, increasing the risk of overfitting and contributing to the wide confidence intervals observed for several coefficient estimates. We prioritized clinical plausibility and limited model complexity. As a sensitivity analysis, we fit a parsimonious adjusted model including age, BMI, procedure type, GH, and Bp, and compared estimates to the full model to assess robustness.

### 3.2. Risk Factors for Subjective Failure

The results of the multivariable logistic regression analysis for subjective failure are shown in Table 4. BMI and preoperative GH were independently associated with the outcome. After adjustment for age, procedure type, preoperative POP-Q measures (Aa, Ba, Ap, Bp, C, D, PB, TVL), and concomitant anterior (cystocele) repair, higher *BMI* was associated with lower odds of subjective failure (adjusted OR, 0.87 per 1 kg/m^2^ increase; 95 confidence interval, 0.77–0.98; *p* = 0.026), whereas larger GH was associated with higher odds of subjective failure (adjusted OR, 1.94 per 1 cm increase; 95 confidence interval, 1.02–3.66; *p* = 0.041). All preoperative POP-Q measures, including Ba, were entered into the multivariable model as part of the prespecified POP-Q adjustment set (domains 1 and 2); Ba was not selected based on its univariable significance. Although point Aa was associated with subjective failure in univariable analysis, it was not statistically significant after adjustment (adjusted OR, 0.67 per 1 cm increase; 95% confidence intervals, 0.42–1.05; *p* = 0.080). No other covariates were significantly associated with subjective failure in the adjusted model (all *p* > 0.05).

### 3.3. Risk Factors for Anatomical Failure

Multivariable logistic regression results for anatomical failure are presented in Table 5. Preoperative GH and Bp were independently associated with the outcome. After adjustment for age, BMI, comorbidities, procedure type, preoperative POP-Q measures, and concomitant anterior (cystocele) repair, larger GH was associated with higher odds of anatomical failure (adjusted OR, 3.55 per 1 cm increase; 95 confidence interval, 1.75–7.58; *p* = 0.001), and higher Bp was similarly associated with increased odds of anatomical failure (adjusted OR, 1.53 per 1 cm increase; 95 confidence interval, 1.14–2.12; *p* = 0.007). POP-Q point Ap and POP-Q point D were also statistically significant in the adjusted model (Ap: adjusted OR, 0.53 per 1 cm increase; 95 confidence intervals, 0.27–0.96; *p* = 0.047; D: adjusted OR, 0.58 per 1 cm increase; 95 confidence intervals, 0.35–0.95; *p* = 0.032).

Hyperthyroidism was associated with markedly higher odds of anatomical failure (adjusted OR, 23.24; 95 confidence intervals, 1.33–623.79; *p* = 0.027); however, given the small number of patients with hyperthyroidism, this estimate should be interpreted cautiously. No other covariates were significantly associated with anatomical failure after adjustment (all *p* > 0.05).

### 3.4. Risk Factors for Composite Failure

Multivariable logistic regression results for the composite outcome are presented in Table 6. Preoperative Bp and GH were independently associated with the outcome. After adjustment for age, BMI, procedure type, preoperative POP-Q measures, and concomitant anterior (cystocele) repair, higher Bp was associated with increased odds of the composite outcome (adjusted OR, 1.31 per 1 cm increase; 95 confidence interval, 1.03–1.70; *p* = 0.031), and larger *GH* was similarly associated with increased odds (adjusted OR, 1.79 per 1 cm increase; 95% confidence interval, 1.02–3.12; *p* = 0.040). POP-Q point Ap demonstrated a borderline association after adjustment (adjusted OR, 0.66 per 1 cm increase; 95% confidence intervals, 0.41–1.02; *p* = 0.074). No other covariates were significantly associated with the composite outcome in the adjusted model (all *p* > 0.05).

### 3.5. Predictive Models

Prediction models were developed to estimate the probability of subjective, anatomical, and composite failure after primary POP surgery. Candidate predictors reflected routinely collected clinical variables (demographic characteristics, surgical approach, and preoperative POP-Q measures) that are available for perioperative risk stratification. Models were trained in Python using penalized logistic regression, random forest, and gradient boosting (LightGBM) and internally validated using bootstrap optimism correction (500 resamples). Model performance was summarized using AUC and PR-AUC for discrimination, Brier score for overall accuracy, and calibration measures (intercept/slope and calibration plots). Given the modest event rates, PR-AUC was emphasized as a complementary measure to AUC.

### 3.6. Optimism-Corrected Prediction Performance

All performance estimates reflect bootstrap optimism correction as described in the Methods section and therefore represent internally validated performance rather than apparent (in-sample) estimates. Calibration-in-the-large (intercept), as shown in Table 7, reflects whether predictions are systematically too high or too low; values far from 0 indicate overall miscalibration. The calibration slope reflects whether predicted risks are too extreme (<1) or too moderate (>1). In this cohort, several models showed substantial intercept deviations, indicating that raw predicted probabilities would require recalibration before clinical use despite strong discrimination.

### 3.7. Model Explainability Using SHAP (Global Feature Attribution)

To improve the interpretability of the machine learning models, we summarized global feature contributions using Shapley additive explanations (SHAP). SHAP values quantify each predictor’s marginal contribution to the model output for each individual, allowing aggregation into global importance (mean absolute SHAP) and visualization of effect directionality (beeswarm plots). Because SHAPs are inherently model-specific and not causal, results are interpreted as patterns of model reliance rather than causal effects.

### 3.8. SHAP Values for Subjective, Anatomical and Composite Failure

**Figure 1:**
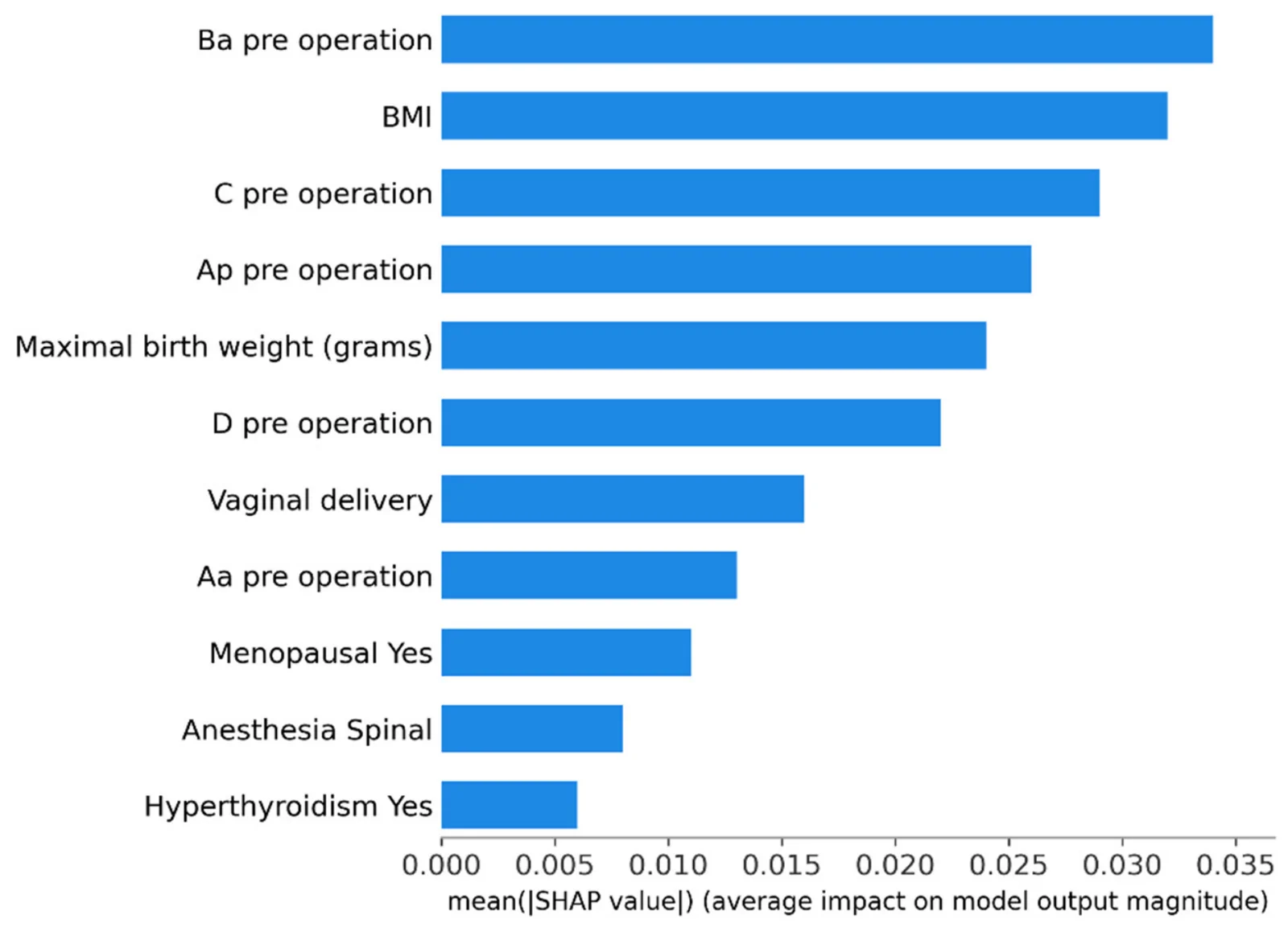
Global SHAP feature importance for subjective failure (random forest). Bar plot shows mean absolute SHAP values across all observations; larger values indicate greater overall contribution to the model prediction.

**Figure 2:**
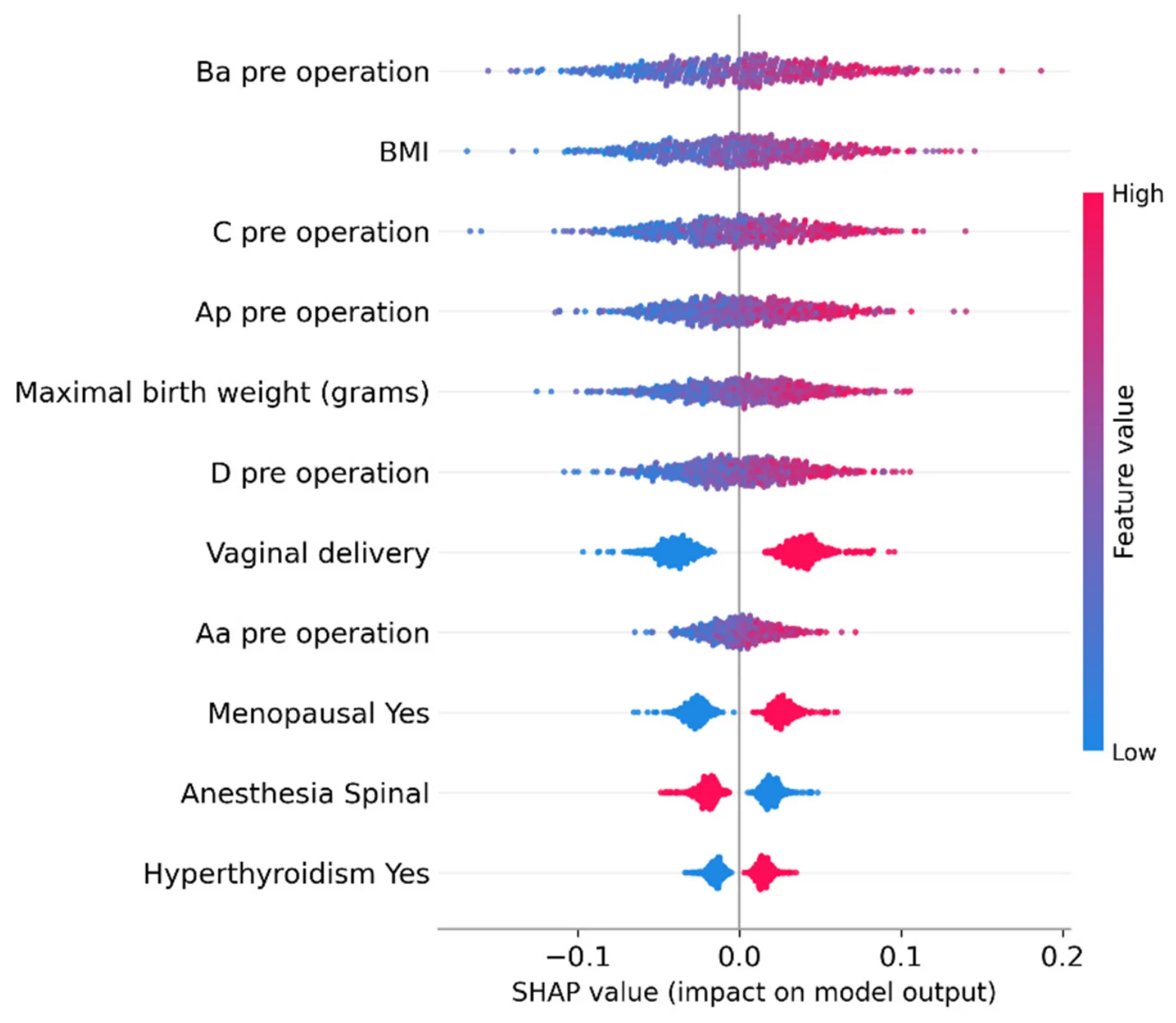
SHAP beeswarm for subjective failure (random forest). Each point represents a patient; x-axis is the SHAP value (impact on predicted probability of failure), and color encodes the feature value (high vs. low).

Table 8 summarizes the most influential predictors in the Subjective failure model, ranked by mean absolute SHAP value (mean(|SHAP|)). Mean(|SHAP|) reflects the average magnitude of each feature’s contribution to the model’s predictions across all patients, irrespective of direction (i.e., it indicates importance, not whether the feature increases or decreases risk). The leading predictors were preoperative prolapse measurements, particularly Ba (0.034) and C (0.029), together with BMI (0.031) and Ap (0.026). Maximal birth weight (0.024) and preoperative D (0.022) also contributed meaningfully, while vaginal delivery history showed a moderate contribution (0.016). Overall, the prominence of multiple preoperative POP-Q points (Ba, C, Ap, D, and Aa) indicates that baseline anatomic severity drives a substantial portion of the model’s predictive signal, with patient anthropometrics (BMI) and obstetric history (maximal birth weight, vaginal delivery) providing additional information. In contrast, Aa (0.009), menopausal status (0.008), and spinal anesthesia (0.002) had relatively small average impact on predictions, suggesting limited incremental value beyond the core anatomic and anthropometric predictors. Directionality and patient-specific effects should be interpreted using SHAP dependence plots or individual explanation plots rather than this global ranking alone (Figure 1 and Figure 2). Notably, genital hiatus (GH), which was the primary independent predictor in logistic regression analysis, does not appear among the top SHAP features in the machine learning model. This discrepancy reflects the distinct mechanisms by which these two approaches allocate predictive weight—logistic regression adjusts for collinear predictors sequentially, whereas machine learning models may distribute the predictive signal of correlated variables across multiple features. These rankings are therefore exploratory and should not be interpreted as definitive measures of clinical importance or as contradicting the logistic regression findings.

**Figure 3:**
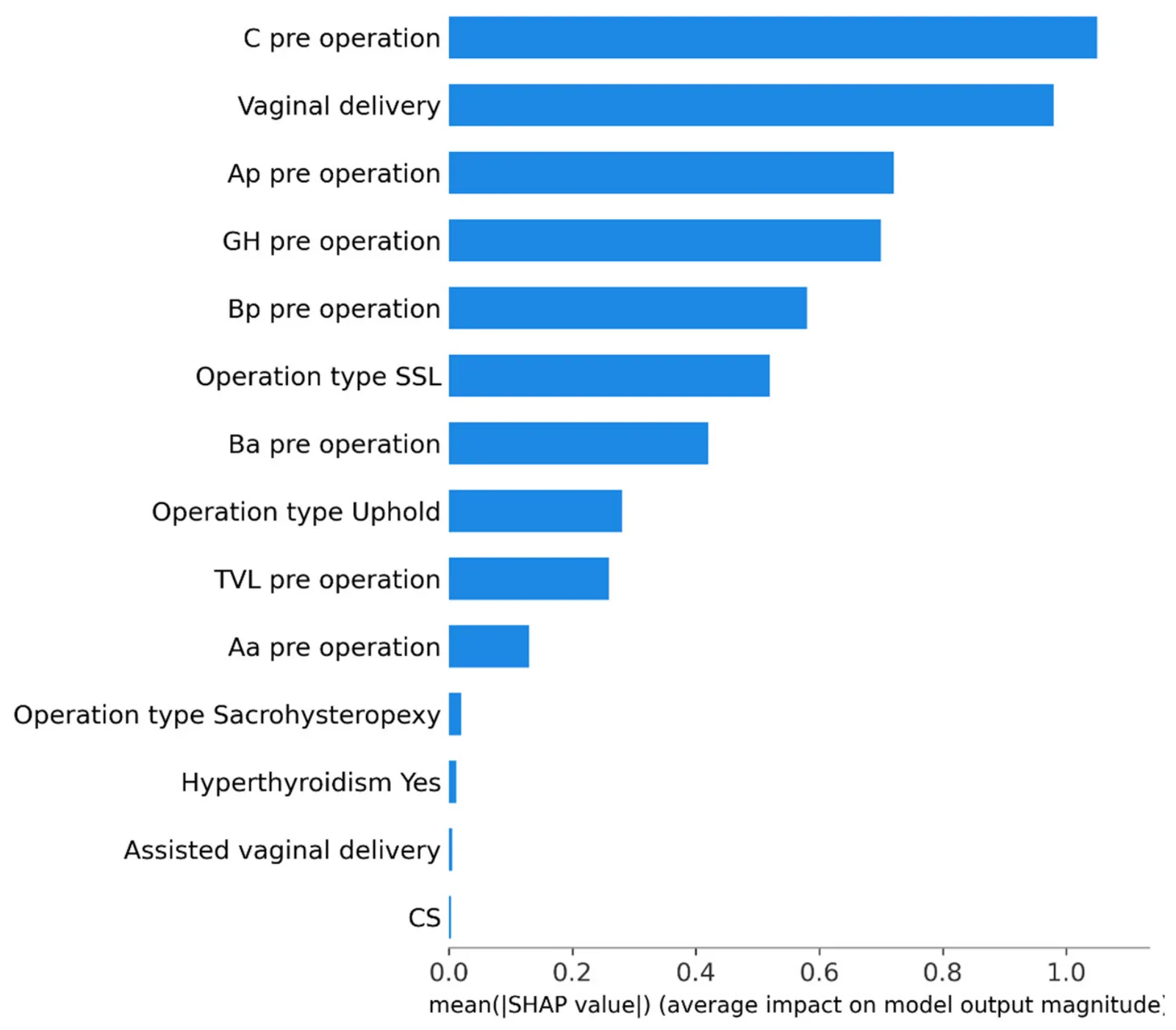
Global SHAP feature importance for anatomical failure (LightGBM). Bar plot shows mean absolute SHAP values across all observations; larger values indicate greater overall contribution to the model prediction.

**Figure 4:**
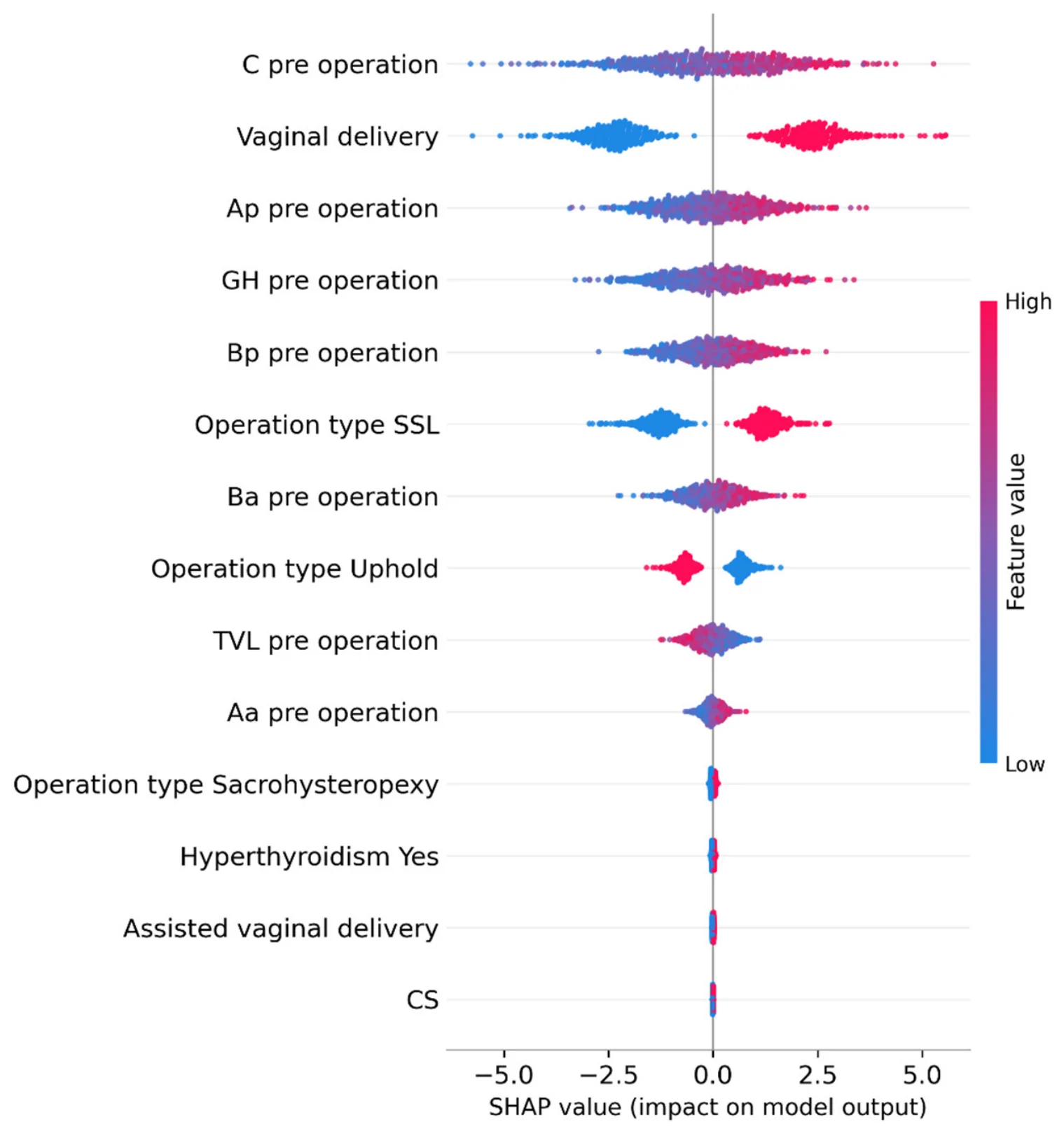
SHAP beeswarm for anatomical failure (LightGBM). Each point represents a patient; x-axis is the SHAP value (impact on predicted probability of failure), and color encodes the feature value (high vs. low).

Table 9 summarizes the most influential predictors in the anatomical failure model, ranked by mean absolute SHAP value (mean(|SHAP|)). The dominant predictor was the preoperative C point (1.109), followed by vaginal delivery history (0.990). Additional high-impact anatomic measures included Ap (0.720), genital hiatus (GH; 0.711), perineal body (Bp; 0.572), Ba (0.447), total vaginal length (TVL; 0.282), and Aa (0.139), underscoring that baseline pelvic support and compartment-specific severity drive a substantial share of the model’s predictive signal. Notably, the surgical approach also contributed meaningfully: operation type SSL (0.561) and Uphold (0.292) ranked among the top predictors, suggesting that procedural selection provides incremental information beyond preoperative anatomy and obstetric history. Directionality and patient-specific effects should be interpreted using SHAP dependence plots or individual explanation plots rather than this global ranking alone (Figure 3 and Figure 4).

**Figure 5:**
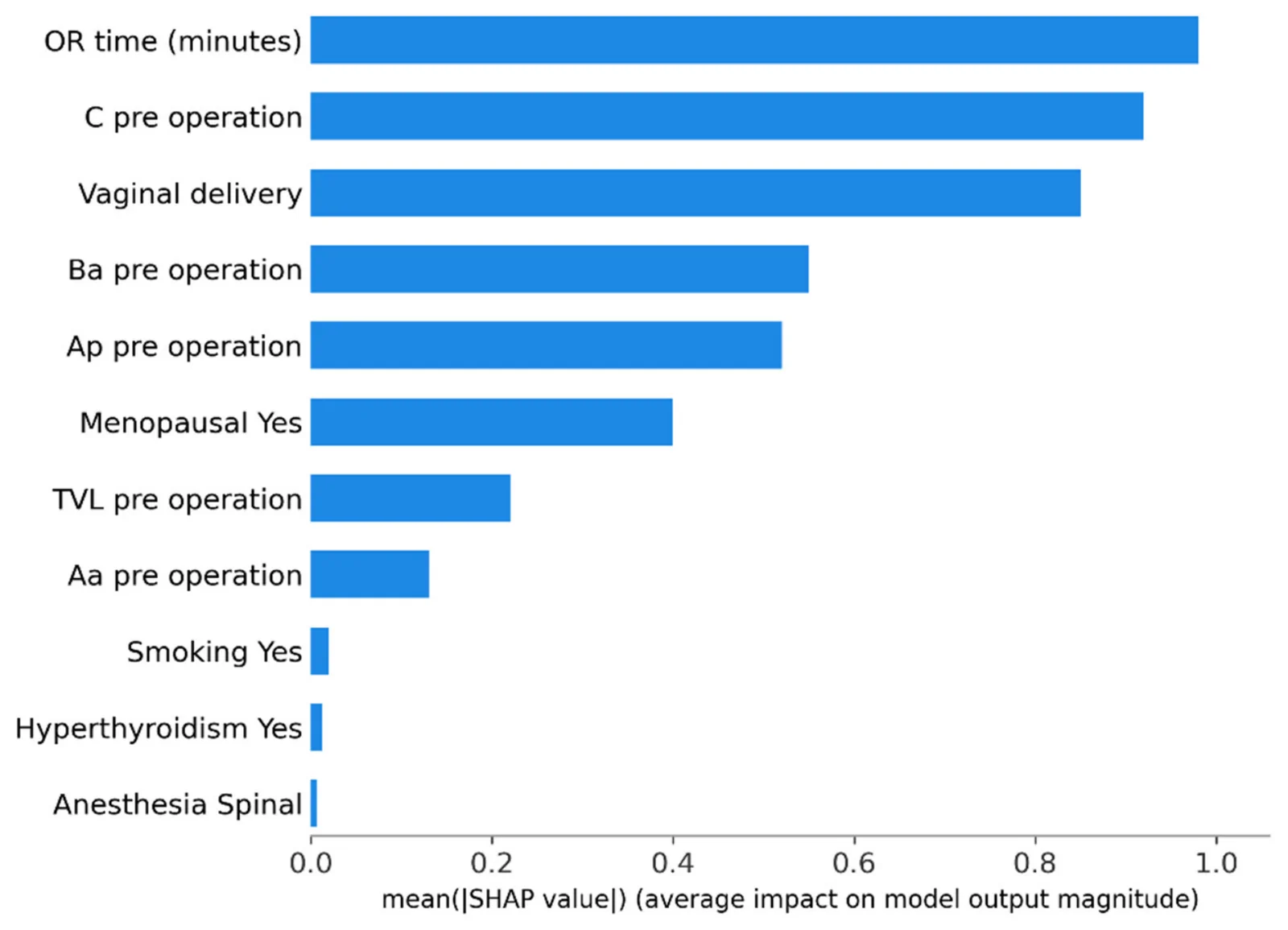
Global SHAP feature importance for composite outcome failure (LightGBM). Bar plot shows mean absolute SHAP values across all observations; larger values indicate greater overall contribution to the model prediction.

**Figure 6:**
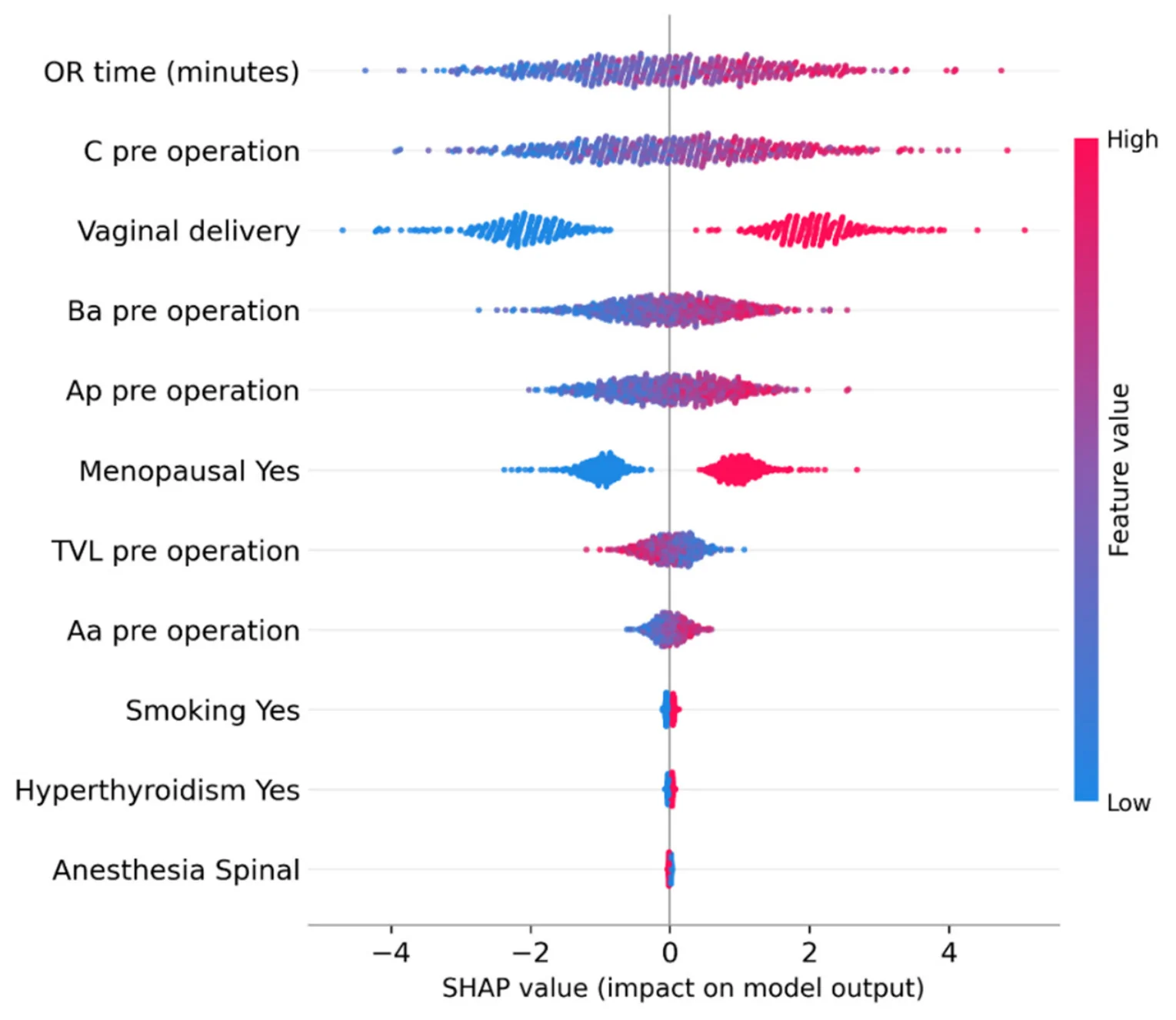
SHAP beeswarm for composite outcome failure (LightGBM). Each point represents a patient; x-axis is the SHAP value (impact on predicted probability of failure), and color encodes the feature value (high vs. low).

Table 10 summarizes the most influential predictors in the Composite outcome model, ranked by mean absolute SHAP value (mean(|SHAP|)). The most influential variable was operative time (OR time, minutes; 0.989), followed by the preoperative C point (0.951) and vaginal delivery history (0.843). Key anatomic measures, Ba (0.544), Ap (0.538), TVL (0.235), and Aa (0.135), also ranked among the top predictors, indicating that baseline pelvic support remains a major driver of the model’s predictive signal. Menopausal status contributed a moderate additional impact (0.404), suggesting incremental information beyond anatomy and operative factors. In contrast, smoking status and spinal anesthesia had mean(|SHAP|) values of 0.000, indicating a negligible contribution in this model and dataset. Directionality and patient-specific effects should be interpreted using SHAP dependence plots or individual explanation plots rather than this global ranking alone (Figure 5 and Figure 6).

Across endpoints, SHAPs indicated that model predictions were driven primarily by baseline pelvic support parameters (POP-Q) and select clinical/operative factors, consistent with the clinical premise that preoperative anatomy encodes substantial recurrence risk. Global importance rankings (mean absolute SHAP) were dominated by POP-Q landmarks reflecting apical support and vaginal caliber (e.g., C, GH/TVL when included), with additional contributions from compartment-specific descent measures and obstetric history variables. Importantly, SHAP patterns represent how the fitted model allocates predictive weight in this cohort and should not be interpreted as causal effects.

Across endpoints, global SHAP rankings indicated that baseline POP-Q parameters (apical position C, genital hiatus, and posterior compartment measures) dominated model predictions, suggesting that preoperative anatomic severity encodes substantial recurrence risk. Operative time emerged as an important feature for the composite model, which may function as a proxy for surgical complexity rather than a causal driver.

## 4. Discussion

In this retrospective cohort of women undergoing primary uterine-preserving POP surgery, we identified clinically interpretable risk factors for failure across subjective, anatomical, and composite endpoints and embedded internally validated prediction outputs generated in Python. Across endpoints, larger genital hiatus emerged as a consistent risk factor and posterior compartment descent was independently associated with anatomical and composite failure. These findings suggest that baseline pelvic floor metrics—particularly hiatal dimensions and posterior support—may be relevant for preoperative risk stratification; however, given the retrospective single-center design and modest event counts, these associations should be considered preliminary and require prospective external validation before informing routine counseling or surgical planning.

Large genital hiatus and posterior compartment defects may reflect significant obstetric trauma, potentially increasing the risk of subsequent POP. These findings may also indicate underlying connective tissue weakness, which could predispose affected women to higher rates of surgical failure.

Internally validated prediction models demonstrated strong optimism-corrected discrimination for all three endpoints. However, calibration performance was suboptimal across several models: calibration intercepts and slopes deviated notably from ideal values, indicating that predicted probabilities may systematically overestimate or underestimate true event risk. This miscalibration represents a critical limitation for clinical translation—a poorly calibrated model cannot reliably convey accurate absolute risk estimates to patients, limiting its utility for shared decision-making. Accordingly, statements regarding readiness for perioperative decision-support are premature, and the current findings should be interpreted as demonstrating preliminary predictive performance only. Future work must prioritize recalibration, external validation in independent cohorts, and evaluation of clinical utility through formal decision-curve analysis before these models are considered suitable for bedside implementation.

Strengths of this study include standardized POP-Q assessment, evaluation of multiple clinically relevant endpoints, and internal validation of prediction performance. Limitations include the single-center design, modest event counts (particularly for anatomical failure), and absence of external validation. The calibration performance of several models was suboptimal, and these models are not suitable for direct clinical use without recalibration. Outcomes were analyzed as binary variables without accounting for variable follow-up durations, which limits the interpretation of absolute failure rates and precludes time-to-event analysis. Additionally, the inclusion of statistically significant baseline comparisons as candidate predictors introduces a degree of outcome-driven variable selection that may inflate apparent model performance. Although the overall sample size was relatively small, the population was intentionally restricted to primary uterine-preserving procedures. This clinical design focused on a specific patient population and enabled more coherent estimation of recurrence risk within a defined surgical context. Importantly, the identified risk patterns were consistent with established biomechanical mechanisms and previously reported clinical associations, supporting the representativeness and face validity of the findings. The internally validated prediction performance further suggests that, despite modest event counts, the dataset was sufficient to develop stable and clinically meaningful prognostic models. Thus, rather than reflecting a limitation alone, the focused cohort facilitated a methodologically rigorous transition from traditional association testing to individualized risk prediction within a real-world surgical population.

In this cohort of women undergoing primary uterine-preserving POP repair, recurrence was evaluated across three clinically relevant endpoints: subjective, anatomical, and composite. These endpoints reflect related but not identical clinical outcomes. Enlarged genital hiatus and posterior compartment descent were consistent and clinically interpretable predictors across outcomes, whereas most demographic and obstetric factors showed limited predictive value. Compared with previous studies, the present findings emphasize the limited predictive contribution of commonly used clinical variables when considered in isolation, and the advantage of combining multiple parameters into a single prediction framework. These results support the feasibility of machine learning-based risk stratification in urogynecological care; however, the current findings represent preliminary, internally validated evidence only. Prospective external validation, recalibration, and formal assessment of clinical impact are required before these models can be recommended for routine clinical use.

## Figures and Tables

**Table 1:** Baseline characteristics by subjective failure.

Dependent: Subjective Failure	No (n = 233)	Yes (n = 36)	*p*
Age at operation	60.4 (9.6)	60.4 (13.1)	0.979
BMI	26.1 (4.1)	24.5 (3.1)	0.029
Parity	5.2 (3.0)	4.5 (2.7)	0.173
Vaginal delivery	5.0 (3.0)	4.4 (2.7)	0.270
Cesarean delivery	0.2 (0.5)	0.1 (0.3)	0.285
Assisted vaginal delivery	19 (8.3)	4 (11.1)	0.805
Maximal birth weight (grams)	3674.9 (517.5)	3657.6 (867.9)	0.871
Smoking	11 (4.8)	0 (0.0)	0.375
Menopausal	52 (22.7)	13 (36.1)	0.126
Sexually active	145 (88.4)	24 (92.3)	0.801
Comorbidities			
CHF	1 (0.4)	1 (2.8)	0.628
Hyperthyroidism	1 (0.4)	2 (5.6)	0.061
Arrythmia	3 (1.3)	1 (2.8)	1.000
Atrial fibrillation	3 (1.3)	1 (2.8)	1.000
Hypothyroidism	26 (11.2)	5 (13.9)	0.844
Hypertension	34 (14.6)	7 (19.4)	0.614
DM	24 (10.3)	1 (2.8)	0.255
Prior pelvic surgery	20 (8.6)	2 (5.6)	0.766
Previous incontinence surgery	1 (0.4)	0 (0.0)	1.000
POP-Q			
Aa pre-op	2.4 (1.3)	1.9 (1.7)	0.036
Ba pre-op	3.7 (2.0)	3.8 (3.2)	0.628
C pre-op	0.1 (3.5)	0.3 (4.9)	0.689
GH pre-op	4.9 (0.7)	5.2 (1.2)	0.051
PB pre-op	3.0 (0.3)	3.1 (0.2)	0.359
TVL pre-op	9.3 (0.6)	9.5 (0.8)	0.156
Ap pre-op	−0.7 (1.3)	−0.5 (1.9)	0.323
Bp pre-op	−0.4 (2.2)	0.2 (3.5)	0.172
D pre-op	−4.6 (1.6)	−4.9 (1.8)	0.366
Pre-op hemoglobin	13.2 (1.1)	13.3 (1.3)	0.623
Operation type			0.695
LUSLS	119 (51.1)	18 (50.0)	
Sacrohysteropexy	24 (10.3)	4 (11.1)	
SSLF	56 (24.0)	11 (30.6)	
Uphold	34 (14.6)	3 (8.3)	
Concomitant procedures			
Mid-urethral sling	120 (51.5)	19 (52.8)	1.000
Anterior repair	188 (80.7)	29 (80.6)	1.000
Posterior repair	178 (76.4)	29 (80.6)	0.735
Cervical amputation	24 (10.4)	2 (5.6)	0.539
Anesthesia			0.221
General	217 (97.3)	33 (91.7)	
Spinal	6 (2.7)	3 (8.3)	
OR time (minutes)	122.3 (60.6)	117.7 (81.4)	0.716

Note: BMI, body mass index; CHF, chronic heart failure; DM, diabetes melitus; POP-Q, pelvic organ prolapse quantification system; LUSLS, laparoscopic uterosacral ligament suspension; SSLF, sacrospinous ligament fixation; OR, operating room.

**Table 2:** Baseline characteristics by anatomical failure.

Dependent: Anatomical Failure	No (n = 244)	Yes (n = 24)	*p*
Age at operation	60.4 (10.0)	60.6 (11.6)	0.935
BMI	25.8 (4.1)	26.3 (4.1)	0.557
Parity	5.0 (2.9)	5.8 (3.7)	0.229
Vaginal delivery	4.8 (2.9)	5.6 (3.7)	0.202
Cesarean delivery	0.2 (0.5)	0.2 (0.4)	0.963
Assisted vaginal delivery	22 (9.1)	1 (4.2)	0.658
Maximal birth weight (grams)	3672.5 (564.9)	3697.1 (680.0)	0.843
Smoking	11 (4.5)	0 (0.0)	0.597
Menopausal	57 (23.8)	8 (33.3)	0.429
Sexually active	155 (89.6)	13 (81.2)	0.548
Comorbidities			
CHF	1 (0.4)	1 (4.2)	0.425
Hyperthyroidism	1 (0.4)	2 (8.3)	0.012
Arrythmia	4 (1.6)	0 (0.0)	1.000
Atrial fibrillation	3 (1.2)	1 (4.2)	0.802
Hypothyroidism	29 (11.9)	2 (8.3)	0.853
Hypertension	35 (14.3)	6 (25.0)	0.277
DM	23 (9.4)	2 (8.3)	1.000
Prior pelvic surgery	20 (8.2)	2 (8.3)	1.000
Previous incontinence surgery	1 (0.4)	0 (0.0)	1.000
POP-Q			
Aa pre-op	2.3 (1.3)	1.9 (1.8)	0.173
Ba pre-op	3.6 (1.9)	4.7 (4.0)	0.015
C pre-op	−0.1 (3.3)	2.1 (5.8)	0.003
GH pre-op	4.9 (0.7)	5.7 (1.2)	<0.001
PB pre-op	3.0 (0.3)	3.0 (0.3)	0.817
TVL pre-op	9.3 (0.6)	9.8 (1.2)	<0.001
Ap pre-op	−0.7 (1.3)	0.0 (2.1)	0.010
Bp pre-op	−0.6 (1.8)	2.1 (4.9)	<0.001
D pre-op	−4.7 (1.6)	−4.3 (2.3)	0.320
Pre-op hemoglobin	13.2 (1.1)	13.2 (1.6)	0.931
Operation type			0.054
LUSLS	131 (53.7)	6 (25.0)	
Sacrohysteropexy	25 (10.2)	3 (12.5)	
SSL	57 (23.4)	10 (41.7)	
Uphold	31 (12.7)	5 (20.8)	
Concomitant procedures			
Mid-urethral sling	127 (52.0)	11 (45.8)	0.713
Anterior repair	201 (82.4)	16 (66.7)	0.110
Posterior repair	190 (77.9)	17 (70.8)	0.597
Cervical amputation	24 (10.0)	2 (8.3)	1.000
Anesthesia			0.436
General	228 (97.0)	22 (91.7)	
Spinal	7 (3.0)	2 (8.3)	
OR time (minutes)	121.0 (62.9)	130.2 (71.7)	0.557

Data presented as [mean ± SD] or [n (%)]. Note: BMI, body mass index; CHF, chronic heart failure; DM, diabetes melitus; POP-Q, pelvic organ prolapse quantification system; LUSLS, laparoscopic uterosacral ligament suspension; SSL, sacrospinous ligament fixation; OR, operating room.

**Table 3:** Baseline characteristics by composite outcome.

Dependent: Composite Outcome	No (n = 224)	Yes (n = 46)	*p*
Age at operation	60.5 (9.7)	59.7 (11.9)	0.622
BMI	26.0 (4.1)	25.1 (3.6)	0.202
Parity	5.1 (2.9)	4.9 (3.1)	0.665
Vaginal delivery	4.9 (2.9)	4.8 (3.1)	0.840
Cesarean delivery	0.2 (0.5)	0.1 (0.3)	0.428
Assisted vaginal delivery	19 (8.6)	4 (8.7)	1.000
Maximal birth weight (grams)	3687.9 (512.3)	3614.9 (814.5)	0.449
Smoking	11 (5.0)	0 (0.0)	0.257
Menopausal	49 (22.3)	16 (34.8)	0.108
Sexually active	138 (88.5)	32 (91.4)	0.835
Comorbidities			
CHF	1 (0.4)	1 (2.2)	0.764
Hyperthyroidism	1 (0.4)	2 (4.3)	0.127
Arrythmia	3 (1.3)	1 (2.2)	1.000
Atrial fibrillation	3 (1.3)	1 (2.2)	1.000
Hypothyroidism	26 (11.6)	5 (10.9)	1.000
Hypertension	32 (14.3)	9 (19.6)	0.494
DM	23 (10.3)	2 (4.3)	0.326
Prior pelvic surgery	20 (9.0)	2 (4.3)	0.456
Previous incontinence surgery	1 (0.4)	0 (0.0)	1.000
POPQ			
Aa pre-op	2.3 (1.3)	2.1 (1.6)	0.256
Ba pre-op	3.5 (1.9)	4.3 (3.2)	0.046
C pre-op	−0.1 (3.3)	0.9 (5.1)	0.080
GH pre-op	4.9 (0.7)	5.2 (1.1)	0.005
PB pre-op	3.0 (0.3)	3.1 (0.3)	0.487
TVL pre-op	9.3 (0.6)	9.6 (0.9)	0.007
Ap pre-op	−0.7 (1.3)	−0.5 (1.8)	0.393
Bp pre-op	−0.6 (1.9)	0.6 (4.0)	0.002
D pre-op	−4.6 (1.6)	−4.7 (1.8)	0.931
Pre-op hemoglobin	13.2 (1.1)	13.3 (1.2)	0.732
Operation type			0.601
LUSLS	117 (52.2)	20 (43.5)	
Sacrohysteropexy	24 (10.7)	4 (8.7)	
SSLF	53 (23.7)	14 (30.4)	
Uphold	30 (13.4)	8 (17.4)	
Concomitant procedures			
Mid-urethral sling	114 (50.9)	25 (54.3)	0.791
Anterior repair	183 (81.7)	34 (73.9)	0.314
Posterior repair	174 (77.7)	33 (71.7)	0.499
Cervical amputation	23 (10.4)	3 (6.5)	0.592
Anesthesia			0.416
General	209 (97.2)	43 (93.5)	
Spinal	6 (2.8)	3 (6.5)	
OR time (minutes)	123.1 (61.1)	110.3 (75.6)	0.270

Data presented as [mean ± SD] or [n (%)]. Note: BMI, body mass index, CHF, chronic heart failure, DM, diabetes melitus, POPQ, pelvic organ prolapse quantification system, LUSLS, laparoscopic uterosacral ligament suspension, SSLF, sacrospinous ligament fixation, OR, operating room.

**Table 4:** Logistic regression results for subjective failure (univariable and multivariable).

Dependent: Subjective Failure	No (n = 233)	Yes (n = 33)	OR (Univariable)	OR (Multivariable)
Age at operation	60.4 (9.6)	60.4 (13.1)	1.00 (0.97–1.04, *p* = 0.979)	1.01 (0.96–1.06, *p* = 0.735)
BMI	26.1 (4.1)	24.5 (3.1)	0.89 (0.80–0.98, *p* = 0.030)	0.87 (0.77–0.98, *p* = 0.026)
Operation type				
LUSLS	119 (86.9)	18 (13.1)	-	-
Sacrohysteropexy	24 (85.7)	4 (14.3)	1.10 (0.30–3.28, *p* = 0.871)	1.34 (0.25–5.90, *p* = 0.715)
SSLF	56 (83.6)	11 (16.4)	1.30 (0.56–2.90, *p* = 0.530)	1.33 (0.45–3.77, *p* = 0.600)
Uphold	34 (91.9)	3 (8.1)	0.58 (0.13–1.85, *p* = 0.409)	0.54 (0.06–4.53, *p* = 0.567)
POPQ				
Aa pre op	2.4 (1.3)	1.9 (1.7)	0.79 (0.63–1.00, *p* = 0.040)	0.67 (0.42–1.05, *p* = 0.080)
Ba pre-op	3.7 (2.0)	3.8 (3.2)	1.04 (0.88–1.21, *p* = 0.626)	1.32 (0.87–2.03, *p* = 0.196)
C pre-op	0.1 (3.5)	0.3 (4.9)	1.02 (0.92–1.12, *p* = 0.688)	0.87 (0.69–1.09, *p* = 0.249)
GH pre-op	4.9 (0.7)	5.2 (1.2)	1.45 (0.98–2.11, *p* = 0.057)	1.94 (1.02–3.66, *p* = 0.041)
PB pre-op	3.0 (0.3)	3.1 (0.2)	1.81 (0.54–6.58, *p* = 0.357)	1.84 (0.47–7.96, *p* = 0.400)
TVL pre-op	9.3 (0.6)	9.5 (0.8)	1.39 (0.84–2.21, *p* = 0.946)	1.03 (0.45–2.19, *p* = 0.166)
Ap pre-op	−0.7 (1.3)	−0.5 (1.9)	1.13 (0.88–1.43, *p* = 0.323)	0.95 (0.57–1.61, *p* = 0.860)
Bp pre-op	−0.4 (2.2)	0.2 (3.5)	1.09 (0.95–1.22, *p* = 0.180)	1.04 (0.76–1.37, *p* = 0.816)
D pre-op	−4.6 (1.6)	−4.9 (1.8)	0.89 (0.68–1.12, *p* = 0.357)	0.81 (0.55–1.15, *p* = 0.265)
Anterior repair	188 (86.6)	29 (13.4)	0.99 (0.43–2.59, *p* = 0.985)	0.68 (0.15–3.51, *p* = 0.628)

Data presented as [mean ± SD] or [n (%)]. Note: BMI, body mass index; POPQ, pelvic organ prolapse quantification system; LUSLS, laparoscopic uterosacral ligament suspension; SSLF, sacrospinous ligament fixation.

**Table 5:** Logistic regression results for anatomical failure (univariable and multivariable).

Dependent: Anatomical Failure	No	Yes	OR (Univariable)	OR (Multivariable)
Age at operation	60.4 (10.0)	60.6 (11.6)	1.00 (0.96–1.05, *p* = 0.935)	1.00 (0.94–1.06, *p* = 0.915)
BMI	25.8 (4.1)	26.3 (4.1)	1.03 (0.93–1.13, *p* = 0.556)	0.98 (0.84–1.12, *p* = 0.787)
Hyperthyroidism (Yes)	1 (33.3)	2 (66.7)	22.09 (2.04–486.24, *p* = 0.013)	23.24 (1.33–623.79, *p* = 0.027)
POPQ				
Aa pre-op	2.3 (1.3)	1.9 (1.8)	0.83 (0.64–1.11, *p* = 0.177)	1.36 (0.73–2.79, *p* = 0.359)
Ba pre-op	3.6 (1.9)	4.7 (4.0)	1.22 (1.03–1.45, *p* = 0.018)	0.62 (0.34–1.08, *p* = 0.102)
C pre-op	−0.1 (3.3)	2.1 (5.8)	1.15 (1.04–1.28, *p* = 0.005)	1.17 (0.88–1.56, *p* = 0.287)
GH pre-op	4.9 (0.7)	5.7 (1.2)	2.45 (1.61–3.91, *p* < 0.001)	3.55 (1.75–7.58, *p* = 0.001)
PB pre-op	3.0 (0.3)	3.0 (0.3)	1.18 (0.31–5.21, *p* = 0.816)	0.84 (0.16–5.14, *p* = 0.840)
TVL pre-op	9.3 (0.6)	9.8 (1.2)	2.16 (1.31–3.76, *p* = 0.003)	1.89 (0.76–4.79, *p* = 0.163)
Ap pre-op	−0.7 (1.3)	0.0 (2.1)	1.41 (1.07–1.83, *p* = 0.012)	0.53 (0.27–0.96, *p* = 0.047)
Bp pre-op	−0.6 (1.8)	2.1 (4.9)	1.31 (1.16–1.50, *p* < 0.001)	1.53 (1.14–2.12, *p* = 0.007)
D pre-op	−4.7 (1.6)	−4.3 (2.3)	1.11 (0.87–1.36, *p* = 0.323)	0.58 (0.35–0.95, *p* = 0.032)
Operation type				
LUSLS	131 (95.6)	6 (4.4)	-	-
Sacrohysteropexy	25 (89.3)	3 (10.7)	2.62 (0.53–10.64, *p* = 0.193)	1.34 (0.12–10.66, *p* = 0.796)
SSLF	57 (85.1)	10 (14.9)	3.83 (1.36–11.73, *p* = 0.013)	3.35 (0.74–15.86, *p* = 0.117)
Uphold	31 (86.1)	5 (13.9)	3.52 (0.96–12.44, *p* = 0.048)	1.14 (0.08–22.37, *p* = 0.927)
Anterior repair	201 (92.6)	16 (7.4)	0.43 (0.18–1.11, *p* = 0.068)	0.38 (0.05–4.46, *p* = 0.382)

Data presented as [mean ± SD] or [n (%)]. Note: BMI, body mass index; POPQ, pelvic organ prolapse quantification system; LUSLS, laparoscopic uterosacral ligament suspension; SSLF, sacrospinous ligament fixation.

**Table 6:** Logistic regression results for composite outcome failure (univariable and multivariable).

Dependent: Composite Outcome	No	Yes	OR (Univariable)	OR (Multivariable)
Age at operation	60.5 (9.7)	59.7 (11.9)	0.99 (0.96–1.02, *p* = 0.620)	0.99 (0.95–1.03, *p* = 0.543)
BMI	26.0 (4.1)	25.1 (3.6)	0.95 (0.86–1.03, *p* = 0.202)	0.92 (0.83–1.02, *p* = 0.120)
POPQ				
C pre-op	−0.1 (3.3)	0.9 (5.1)	1.08 (0.99–1.17, *p* = 0.082)	0.95 (0.78–1.16, *p* = 0.617)
GH pre-op	4.9 (0.7)	5.2 (1.1)	1.63 (1.14–2.35, *p* = 0.007)	1.79 (1.02–3.12, *p* = 0.040)
PB pre-op	3.0 (0.3)	3.1 (0.3)	1.49 (0.50–4.75, *p* = 0.485)	1.27 (0.38–4.60, *p* = 0.705)
TVL pre-op	9.3 (0.6)	9.6 (0.9)	1.75 (1.12–2.82, *p* = 0.014)	1.20 (0.60–2.37, *p* = 0.598)
Aa pre-op	2.3 (1.3)	2.1 (1.6)	0.88 (0.71–1.11, *p* = 0.257)	0.88 (0.59–1.35, *p* = 0.560)
Ba pre-op	3.5 (1.9)	4.3 (3.2)	1.15 (1.00–1.32, *p* = 0.049)	1.08 (0.74–1.60, *p* = 0.680)
Ap pre-op	−0.7 (1.3)	−0.5 (1.8)	1.10 (0.87–1.37, *p* = 0.393)	0.66 (0.41–1.02, *p* = 0.074)
Bp pre-op	−0.6 (1.9)	0.6 (4.0)	1.17 (1.05–1.31, *p* = 0.006)	1.31 (1.03–1.70, *p* = 0.031)
D pre-op	−4.6 (1.6)	−4.7 (1.8)	0.99 (0.80–1.19, *p* = 0.931)	0.79 (0.56–1.09, *p* = 0.184)
Operation type				
LUSLS	117 (85.4)	20 (14.6)	-	-
Sacrohysteropexy	24 (85.7)	4 (14.3)	0.98 (0.27–2.86, *p* = 0.966)	0.68 (0.12–2.98, *p* = 0.634)
SSLF	53 (79.1)	14 (20.9)	1.55 (0.71–3.28, *p* = 0.259)	1.79 (0.67–4.68, *p* = 0.234)
Uphold	30 (78.9)	8 (21.1)	1.56 (0.60–3.79, *p* = 0.340)	0.67 (0.10–4.74, *p* = 0.679)
Anterior repair	183 (84.3)	34 (15.7)	0.63 (0.31–1.37, *p* = 0.229)	0.49 (0.11–2.40, *p* = 0.346)

Data presented as [mean ± SD] or [n (%)]. Note: BMI, body mass index; POPQ, pelvic organ prolapse quantification system; LUSLS, laparoscopic uterosacral ligament suspension; SSLF, sacrospinous ligament fixation.

**Table 7:** Optimism-corrected prediction performance (summary).

Outcome	Model	AUC	PR-AUC	Brier	Calibration Intercept (Corrected)	Calibration Slope (Corrected)
	LightGBM	0.880 (0.805–0.947)	0.764 (0.651–0.866)	0.053 (0.041–0.066)	−3.586	1.306
Anatomical failure	Penalized logistic regression	0.659 (0.526–0.772)	0.150 (−0.037–0.294)	0.228 (0.218–0.242)	−2.253	1.695
	Random forest	0.807 (0.743–0.856)	0.254 (0.119–0.383)	0.073 (0.066–0.083)	−0.229	0.570
	LightGBM	0.879 (0.821–0.928)	0.787 (0.705–0.855)	0.093 (0.078–0.110)	−3.441	1.853
Composite outcome failure	Penalized logistic regression	0.626 (0.547–0.700)	0.257 (0.139–0.362)	0.231 (0.207–0.264)	−1.539	0.484
	Random forest	0.843 (0.790–0.887)	0.549 (0.464–0.625)	0.119 (0.107–0.133)	0.256	0.796
	LightGBM	0.880 (0.817–0.936)	0.776 (0.683–0.856)	0.067 (0.053–0.082)	−2.879	1.685
Subjective failure	Penalized logistic regression	0.614 (0.523–0.697)	0.246 (0.109–0.374)	0.229 (0.202–0.261)	−1.762	0.480
	Random forest	0.896 (0.836–0.945)	0.774 (0.681–0.853)	0.080 (0.069–0.091)	1.341	3.028

Note. All metrics shown are bootstrap optimism-corrected (500 resamples).

**Table 8:** Top predictors by mean(|SHAP|) for the subjective failure model.

Feature	Mean(|SHAP|)
Ba pre-op	0.034
BMI	0.031
C pre-op	0.029
Ap pre-op	0.026
Maximal birth weight (grams)	0.024
D pre-op	0.022
Vaginal delivery	0.016
Aa pre-op	0.009
Menopausal	0.008
Anesthesia Spinal	0.002

Note: BMI, body mass index.

**Table 9:** Top predictors by mean(|SHAP|) for the anatomical failure model.

Feature	Mean(|SHAP|)
C pre op	1.109
Vaginal delivery	0.990
Ap pre op	0.720
GH pre op	0.711
Bp pre op	0.572
Operation type SSL	0.561
Ba pre op	0.447
Operation type Uphold	0.292
TVL pre op	0.282
Aa pre op	0.139

**Table 10:** Top predictors by mean(|SHAP|) for the composite outcome model.

Feature	Mean(|SHAP|)
OR time (minutes)	0.989
C pre op	0.951
Vaginal delivery	0.843
Ba pre-op	0.544
Ap pre-op	0.538
Menopausal	0.404
TVL pre-op	0.235
Aa pre-op	0.135
Smoking	0.000
Anesthesia Spinal	0.000

## Data Availability

Data is available upon reasonable request from the corresponding author.

## Appendix B. Supplementary Discrimination Curves

The following ROC curves are included to support the primary performance summary.
